# Effect of *Fuzheng Huayu *formula and its actions against liver fibrosis

**DOI:** 10.1186/1749-8546-4-12

**Published:** 2009-06-29

**Authors:** Chenghai Liu, Yiyang Hu, Lieming Xu, Cheng Liu, Ping Liu

**Affiliations:** 1 Institute of Liver Diseases, Shanghai University of Traditional Chinese Medicine Shuguang Hospital, Shanghai 201203, PR China; 2 Key Laboratory of Liver and Kidney Diseases, Ministry of Education, Shanghai University of Traditional Chinese Medicine, Shanghai 201203, PR China

## Abstract

Liver fibrosis is a common histological process to develop into cirrhosis in various chronic liver diseases including chronic hepatitis and fatty liver. Therefore anti-liver fibrosis is very important strategy to treat chronic liver diseases. *Fuzheng Huayu *(FZHY), a preparation containing herbs such as *Radix Salvia Miltiorrhizae, Cordyceps*, *Semen Persicae*, was formulated on the basis of Chinese medicine theory in treating liver fibrosis and was approved. Pharmacological studies and clinical trials demonstrate that FZHY has a significant effect against liver fibrosis and that many of the pharmacological actions are attributable to the effect. This article reviews the effects and actions of FZHY, in particular the effects observed from clinical trials in treating liver fibrosis caused by chronic hepatitis B and the actions on inhibition of hepatic stellate cell activation, protection of hepatocytes and inhibition of hepatic sinusoidal capillarization. This article also reviews the coordinated effects of the constituent herbs of FZHY and the actions of their active compounds such as salvianonic acid B (SA-B) on liver fibrosis.

## Background

Liver fibrosis is characterized by overproduction and irregular deposition of extracellular matrix (ECM) in liver tissues [1], leading to the distortion of hepatic microstructure and liver dysfunction. The structural changes include hepatic sinusoid capillarization, portal area and liver lobule fibrosis and alterations in microvascular structure. The dysfunction is manifested by the deficiency of liver function and portal hypertension. The main causes of liver fibrosis include hepatitis viruses, alcohol, drugs, toxins, schistosome, nonalcoholic steatohepatitis (NASH), cholestasis and autoimmune liver disease. Their persistent insults on the liver activate hepatic stellate cells (HSCs) in the sinusoid, resulting in the imbalance of ECM metabolism. For example, ECM overproduction may cause over deposition in liver and hepatic structure remodeling. Liver fibrosis can progress into liver cirrhosis which causes further hepatocellular dysfunction and increases intrahepatic resistance to blood flow, leading to hepatic insufficiency and portal hypertension. Liver cirrhosis is the seventh leading cause of disease-related death in the United States [2].

Liver fibrosis was considered to be a passive and irreversible process due to the collapse of the hepatic parenchyma and its substitution with ECM components [3]. However, the reversibility of liver fibrosis has now been demonstrated both in patients and animal models [4].

Antifibrotic strategies against liver fibrosis include early intervention or control of etiologies, hepatic inflammation prevention and regulation of hepatic ECM metabolism and stellate cell activation. Viral hepatitis is the most important antecedent factor for liver fibrosis. Tremendous progress has been made in targeted antiviral treatment in recent years. Recent evidence showed that liver fibrosis could regress with effective antiviral treatment. However, even removal of initial fibrotic stimulus such as viruses may slow fibrosis progression but does not stop the progression entirely [5]. Treatment to improve ECM metabolism is still needed for antiviral treatment. Animal experiments suggest that some fibrosis may persist for very long periods after liver injuries, particularly if the remaining collagen is cross-linked by tissue transglutaminase and thus more resistant to metalloproteinase. Efficacy of antiviral treatment is limited in fibrotic patients suffering from viral infection, in particular hepatitis B patients. Patients with lowered viral replication may have hepatic inflammation which can still develop into cirrhosis through fibrosis. In patients with hepatitis C virus, the severity of liver fibrosis is not necessarily correlated with viral loads or viral genotypes affecting the response of antiviral treatment.

From the studies on liver fibrosis in recent decades [6], we understand that the activation of HSC is a crucial cellular change in liver fibrosis [7]. The regulation of the activation of HSCs has been partially elucidated [8]. The fibrogenetic factors including free radicals, ECM environment and cytokines, in particular transforming growth factor beta one (TGF-β1) were only found in recent years. While effective treatment which targets these specific factors is still not ready. Chinese medicine has significantly contributed to antifibrotic treatment.

### Antifibrotic treatment with Chinese medicinal herbs

Although Chinese medicine does not have the concept of liver fibrosis, its does treat chronic liver diseases effectively. Research on liver fibrosis in Chinese medicine has gone through three stages: (1) Clinical exploration (1950s to 1970s). Chinese medicine considers liver fibrosis as *Xietong *(Hypochondriac pain), *Zhengjia *(mass in the abdomen) and *Guzhang *(Tympanites). The basic pathogenesis of liver fibrosis is regarded as deficiency of healthy energy and stagnation of blood and treatment of liver fibrosis is to activate blood stasis and invigorate spleen according to Chinese medicine syndrome differentiation. Some frequently used formulas include *Taohong *decoction consisting of *Semen Persicae *(*Taoren*), *Flos Carthami *(*Honghua*), *Rhizoma Ligustici Chuanxiong *(*Chuanxiong*), *Radix Angelicae Sinensis *(*Danggui*) and *Radix Clematidis *(*Weilingxian*), and *Xiayuxue *decoction consisting of *Radix et Rhizoma Rhei *(*Dahuang*), *Semen Persicae *and *Eupolyphaga seu Opisthoplatia *(*Zhechong*) [9]. (2) Experimental investigation (late 1970s to early 1990s). The efficacy of Chinese medicine against liver fibrosis was investigated with animal experiments. Effective Chinese medicine formulae and herbs include *Qianggan Ruanjian *decoction [10] consisting of *Radix Angelicae Sinensis*, *Radix Paeoniae Alba *(*Baishao*), *Radix Salviae Miltiorrhizae *(*Danshen*), *Radix Curcumae *(*Yujin*), *Herba Patriniae *(*Baijiangcao*), *Fructus Gardeniae *(*Zhizi*), *Radix Rehmanniae Recens *(*Shengdi*), *Rhizoma Atractylodis Macrocephalae *(*Baizhu*), *Radix Astragali *(*Huangqi*), *Fructus Crataegi *(*Shanzha*) and *Herba Artemisiae Scopariae *(*Yinchen*). In particular, the effects of *Radix Salviae Miltiorrhizae *(*Danshen*) and *Semen Persicae *and their extracts, cucurbitacin B, oleanolic acid, glycyrrhizic acid and hanfangchin A against liver fibrosis were investigated extensively. (3) Clinical trials and molecular studies (1990s onwards).

In 2006, the first national guideline on the diagnosis and treatment of liver fibrosis with integrative medicine was published [9]. The efficacy of Chinese medicine formulae against liver fibrosis is being evaluated in multicenter, randomized controlled clinical trials and the molecular actions are also being studied. In particular, *Fuzheng Huayu *(FZHY) formula has been shown to have efficacy on liver fibrosis, post-hepatic cirrhosis and the prevention of hepatic encephalopathy [11-14].

### Effects of FZHY on liver fibrosis

FZHY formula is a complex preparation to treat liver fibrosis. FZHY consists of six Chinese medicinal herbs, namely *Radix Salvia Miltiorrhizae, Cordyceps *(*Chongcao*), *Semen Persicae, Gynostemma Pentaphyllammak *(*Jiaogulan*), *Pollen Pini *(*Songhuafen*), *Fructus Schisandrae Chinensis *(*Wuweizi*) (Table 1).

**Table 1:** Composition of *Fuzheng Huayu *(FZHY)

**Herbal components**	**Daily dose (g/60 kg adult)**
*Radix Salviae Miltiorrhizae*	8.0
*Fermentation Mycelium Powder*	4.0
*Fructus Schisandrae Chinensis*	2.0
*Semen Persicae*	2.0
*Pollen Pini*	2.0
*Gynostemma Pentaphyllammak*	6.0

The effects of FZHY on the decompensated cirrhosis caused by hepatitis B were investigated in clinical studies [12]. Eighty patients were enrolled and randomly assigned to the control and treatment groups (40 patients per group), and received FZHY plus Vitamins B and C, and Vitamins B and C respectively. The results showed that FZHY improved liver function parameters, albumin (Alb) level in particular, while it decreased γ-globin content, enhanced the plasma ratio of branched chain amino acid (BCAA)/aromatic amino acid (AAA), increased urine Hyp excretion, decreased serum laminin (LM) and haluronic acid (HA) level. FZHY also modulated the immune system, for example, it improved CD3+ and CD4+ counts, natural killer cell activity and complement 3 (C3) content.

The effects of FZHY on liver fibrosis caused by chronic hepatitis B were studied in a clinical trial [11,13], in which 95 patients with chronic hepatitis B were randomly assigned to the treatment (63 patients) and control (32 patients) groups, and received FZHY and Dahuang Zhechong Wan respcetively. The liver function and serological fibrotic markers were tested before and after treatment and 12 patients in the treated group were examined with liver biopsy. The results showed that FZHY markedly decreased serum alanine aminotransferase (ALT) activities and total bilirubin. FZHY also significantly improved serum albumin and A/G ratio, lowered serum monamine oxidase activities, tissue inhibitor of metalloproteinase-1 (TIMP-1), type III procollagen (P-III-P) and LM and increased urine Hyp content. The improvement of these markers except TIMP-1 in treatment group was better than those in the control. Liver biopsy of seven out of 12 patients showed significantly decreased fibrosis. The results suggest that FZHY is effective in treating liver fibrosis and inflammation caused by chronic hepatitis B.

**Figure 1:**
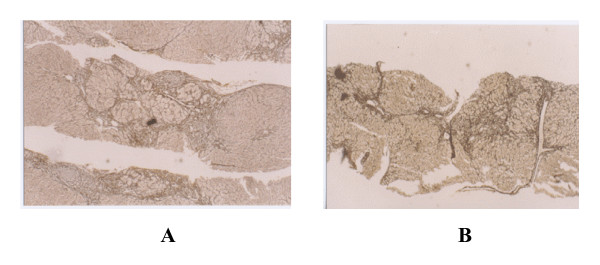
**Effect of FZHY on liver fibrosis in a patient with chronic hepatitis B**. The biopsy liver tissues were stained with VG before (A) and after (B) treatment. × 100.

A multicenter, randomized, double blinded and parallel controlled (Heluoshugan capsule) clinical study on 216 patients with liver fibrosis caused by chronic hepatitis B was carried out to evaluate the safety and efficacy of FZHY [15]. The hepatic histological changes and HBV markers were examined at weeks 0 and 24 of the treatment. Serological parameters (HA, LM, P-III-P, IV-C) and liver function were determined. B ultrasound examination of the spleen and liver was performed at weeks 0, 12 and 24. Blood and urine routines, renal function and ECG were performed before and after treatment. Mean score of fibrotic stage in experiment group after treatment (1.80) decreased significantly (*P *< 0.05) from that before treatment (2.33). There was no significant difference before (2.11) and after (2.14) treatment in the control. There was significant difference in reverse fibrosis rate between the experiment (52%) and control (23.3%) groups in liver biopsy. FZHY inhibited inflammatory activity significantly. Compared to pretreatment, there was a significant decrease in HA, LM, P-III-P and IV-C content after 12 and 24 weeks of treatment. The difference in HA, LM, P-III-P and IV-C content between 12, 24 weeks of treatment and pretreatment in experiment group was significant. The effect, defined as two out of four parameters are more than 30% lower than the baseline, was 72.7% and 27.4% in the experiment and control groups respectively. Improvement in serum Alb, ALT, aspartate aminotransferase (AST) and γ-glutamyl transferase (GGT) was seen in two groups. Marked improvement in GGT and Alb was seen in experiment group (*P *< 0.05). The effective rate of improvement in serum ALT was 72.7% and 59.4% in the experiment and control groups respectively. There was no significant difference in blood and urine routines and ECG before and after treatment. There was also no significant difference in stable rate in ALT and serological parameters for liver fibrosis between the experiment and control groups after 12 weeks' withdrawal. The data show that FZHY is effective in alleviating liver fibrosis caused by chronic hepatitis B (Figure 1) and there was no observable adverse effect.

Recently, we conducted a phase III trial [12] and collectively analyzed the effects of FZHY, on liver fibrosis caused by chronic hepatitis B. The results show that FZHY is effective to treat liver fibrosis caused by chronic hepatitis B, including fibrotic stage S3 with hepatic inflammation, hypochondriac pain and dry mouth. The dynamic pathological changes in liver, the contents of serum Alb, HA and P-III-P, GGT activities, prothrombin time (PT), and blackish complexion, except the serum LN and IV-C, were all found significantly improved after treatment [16].

### Actions of FZHY against liver fibrosis

#### Inhibition of HSC activation

Activation of HSC is a key cellular process of liver fibrosis [17]. Under normal conditions, quiescent HSCs are located in the hepatic perisinusoidal spaces with vitamin A storage in their cytoplsamic lipid droplets. Paracrine activation of HSCs is stimulated by oxidative stress, inflammatory cytokines and endothelial matrix alternation. The activated HSCs release cytokines such as TGF-β and perpetuate the autocrine activation of HSCs. All activated HSCs increase the capabilities of cell proliferation, fibrogenesis and contraction, contributing to the overproduction and accumulation of ECM in liver [18].

**Figure 2:**
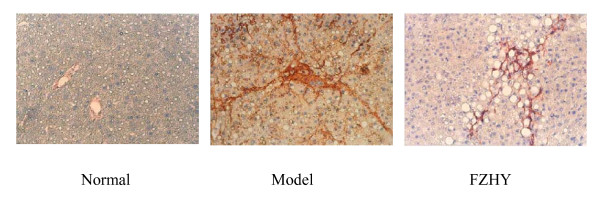
**Effect of FZHY on α-SMA expression in fibrotic liver**. The liver fibrosis was induced with CCl_4 _in rats. Treatment group was administered with FZHY at the start of six-week intoxication, while the control group was given saline; α-SMA expression in liver tissue was determined by immunohistochemical stain. (×100).

We observed the effect of FZHY on α-smooth muscle actin (α-SMA) expression (HSC activation marker) in HSC both *in vivo *and *in vitro*. Liver fibrosis induced by tetrachloride carbon (CCl_4_) or dimethylnitrosamine (DMN) in rats was prophylaxised or treated with FZHY. The results showed that FZHY decreased α-SMA protein expression in the fibrotic liver examined by Western blot and immunohistochemical stain, as well as attenuated ECM deposition in liver (Figure 2).

**Figure 3:**
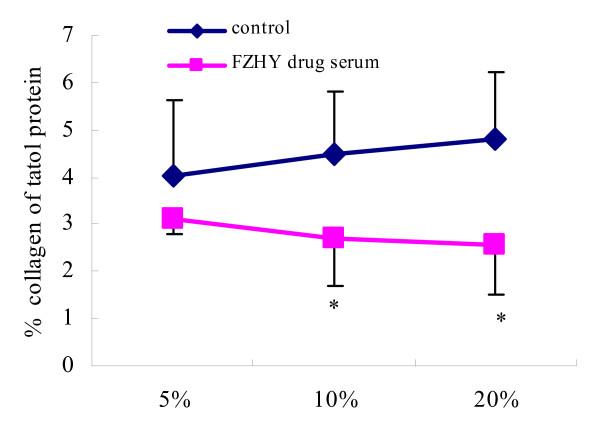
**Effect of FZHY drug serum on collagen secretion from HSC**. The collagen secretion rate was assayed by [^3^H]-Proline incorporation and collagenase digestion; values are expressed as mean ± standard deviation (SD) of three separate experiments and compared by *t *test. *P < 0.05 *vs. *the control. 5%, 10% and 20% mean different concentrations of sera from rats administered with FZHY (FZHY drug serum) or saline (control).

In an *in vitro *study, we isolated and cultured primary HSCs from rats, collected the drug serum [19] from the rats that took FZHY [20] and incubated the HSCs with FZHY drug serum. The results showed that the FZHY drug serum could inhibit α-SMA expression and collagen synthesis in HSCs in a concentration dependent manner (Figure 3). Both *in vivo *and *in vitro *results indicate that FZHY can inhibit HSC activation and that this action is one of the action mechanisms of FZHY against liver fibrosis.

**Figure 4:**
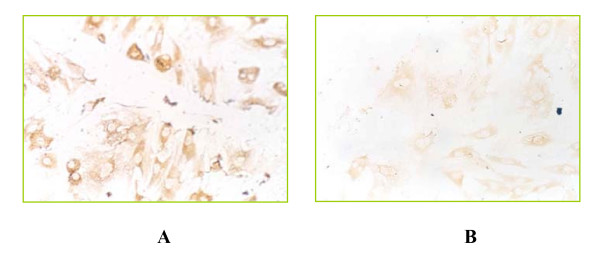
**Effect of FZHY on TGF-β1 expression in activated HSC**. Immunocytochemical stain with anti-TGF-β1 (×200). A: HSC treated with normal rat serum (control); B: HSC treated with FZHY drug serum.

HSCs can be activated by cytokines through autocrine and paracrine processes. Platelet-derived growth factor-BB (PDGF-BB) is a proliferative factor and TGF-β1 is a profibrogenic cytokine; both play pivot roles in HSC activation. We found that the FZHY drug serum inhibited the PDGF-BB-stimulated HSC proliferation and collagen secretion in a dose dependent manner, in particular type I collagen (Col-I) secretion and gene expression, and decreased TGF-β1 expression in activated HSCs (Figure 4). Furthermore, we collected the conditioned medium after treatment of activated HSCs with FZHY drug serum (drug serum treated hepatic stellate cell's conditioned medium, D-HcCM), and incubated the medium with quiescent HSCs (freshly isolated). Results showed that the D-HcCM inhibited HSC spontaneous activation, indicating that FZHY inhibits HSC via down-regulation of an autocrine process [19].

**Figure 5:**
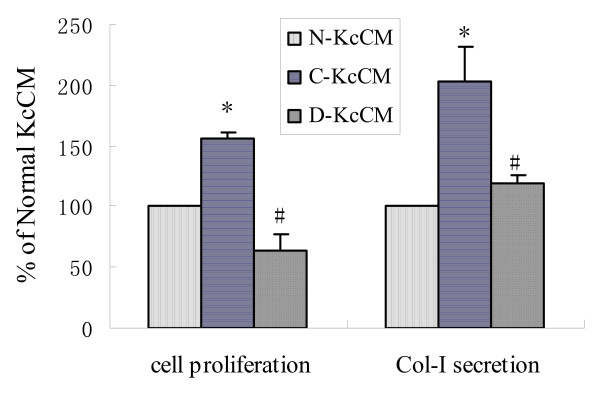
**Effect of FZHY drug serum treated Kuppfer cell conditioned medium on quiescent HSC proliferation and Col-I secretion**. HSC proliferation was assayed with [^3^H]TdR incorporation HSC; type I collagen secretion was measured at HSC supernatants with ELISA. Values are expressed as mean ± standard deviation (SD) of three separate experiments and compared by ANOVA. *P < 0.05, *vs. *N-KcCM, #*P *< 0.05, *vs. *C-KcCM.

Cytokines such as PDGF-BB and TGF-β1 are also secreted by Kuppfer cells at an early stage of liver injury and simulate HSC activation via a paracrine process. We isolated Kuppfer cells from normal and liver-injured rats and incubated the cells with the FZHY drug serum, collected the culture medium as Kuppfer cell conditioned medium (KcCM). We tested the effect of all kinds of conditional media on quiescent HSC. We found that the control medium collected from liver-injured rats had higher concentration of TGF-β1 and PDGF-BB than that in the medium collected from normal rats and increased quiescent HSC cell proliferation and Col-I secretion. FZHY drug serum reduced the TGF-β1 and PDGF contents in Kuppfer cells from liver-injured rats. Moreover, FZHY drug serum decreased HSC proliferation and Col-I secretion in comparison to the cells treated with the control medium. These findings suggest that FZHY can inhibit Kuppfer cell activation and its paracrine effects on HSC activation [21] (Figure 5).

**Figure 6:**
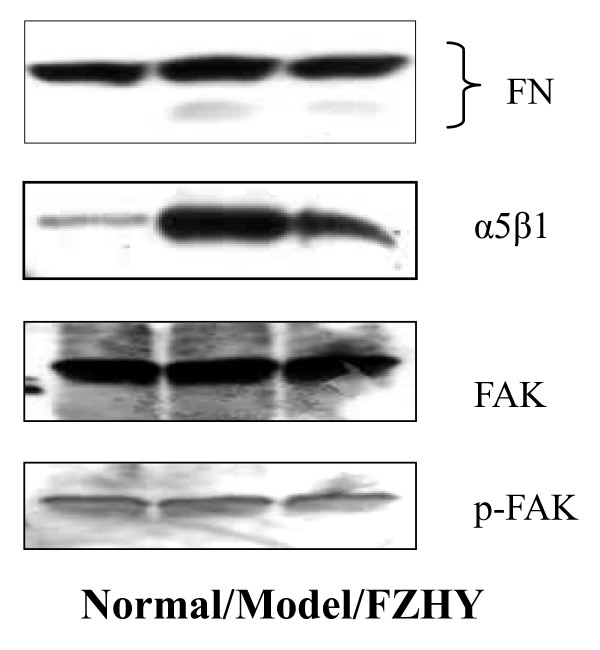
**Effect of FZHY on FN and integrin pathway mediators**. Liver fibrosis was induced by DMN and treated with FZHY. Normal: normal rats; Model, model control; FZHY: FZHY-treated. Protein expression and FAK phosphorylation were checked with Western blot.

Besides cytokines, ECM deposition also stimulates HSC activation, which can regulate cell functions through integrin pathway. We found that at early stage of liver injury in DMN-induced fibrotic rats, hepatic fibronectin (FN) increased and then HSC activated as well as integrin α5β1 expression and focal adhesion kinase (FAK) phosphorylation increased. FZHY decreased FN expression and reduced integrin α5β1 and FAK phosphorylation [22]. We coated plastics with FN and planted primarily isolated HSC on it. Results show that FN stimulates quiescent HSC activation, but FZHY drug serum inhibits FN stimulates HSC activation, and this action was associated with the down-regulation of integrin expression and FAK phosphorylation [23], i.e. FZHY inhibits HSC activation via FN/integrin pathway (Figure 6).

#### Protection of hepatocytes from oxidative stress and apoptosis

Liver injuries, such as hepatocytic inflammatory necrosis and apoptosis, are the precursors of liver fibrosis [24]. Free radicals and oxidative products such as malondialdehyde (MDA) stimulate HSC activation. Liver peroxidation also increases matrix metalloproteinases-2/9 (MMP-2/9), thereby degrading membrane matrix and disrupting hepatic micro-structure and finally activating HSCs. Therefore, liver injury is a bridge between liver inflammation and fibrosis and protecting hepatocytes from oxidative and apoptosis is important in preventing liver fibrosis.

**Figure 7:**
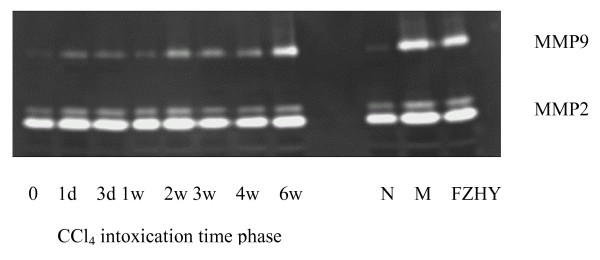
**Dynamic changes of MMP2/9 activity and effect of FZHY on fibrotic liver of CCl_4 _induced rat**. Gelatin zymography. Time phases after CCl_4 _intoxication. N: normal; M: model control; FZHY: FZHY-treated.

CCl_4_-intoxicated rats had features of hepatocytic fatty degeneration, liver steatohepatitis and fibrosis. In our studies [25,26] with CCl_4_-intoxicated rats, FZHY protected hepatocytes from degeneration and necrosis, improved serum liver functionl, inhibited hepatic lipid peroxidation through improved SOD activity and decreased MDA content, and decreased liver hydroproline content for collagen production. We also found that FZHY lowered MMP-2/9 activities in fibrotic liver during continued CCl_4_-intoxication (Figure 7). To confirm FZHY effect on hepatocyte injury, we isolated and cultured the primary cultured hepatocytes from rats, induced cell injury with CCl_4_, and incubated the cells with FZHY drug serum for 48 hours. FZHY did improve the recovery of injured hepatocytes *in vitro*, indicating that FZHY exerts its anti-fibrosis effects through protecting hepatocytes from lipid peroxidation and changes in hepatic micro-structure.

**Figure 8:**
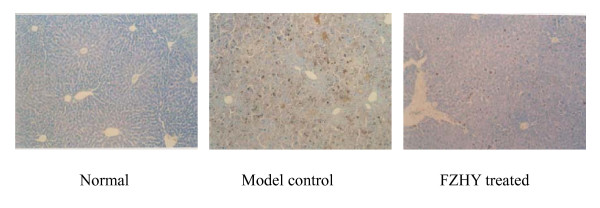
**Effect of FZHY on hepatocytes apoptosis *in vivo***. Acute liver injury with hepatocyte apoptosis was induced by infusion of LPS plus D-GalN for six hours in mice. Treatment group was administered with FZHY two days before LPS intoxication, while the control was given saline. The apoptotic hepatocytes were stained with TUNEL. (×100).

**Figure 9:**
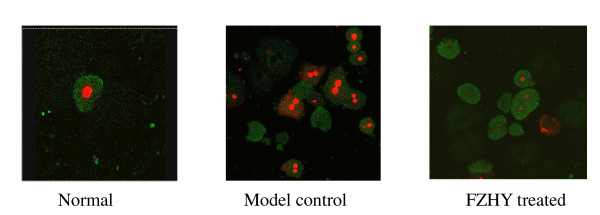
**Effect of FZHY drug serum on apoptosis in hepatocytes *in vitro***. Primary hepatocytes were isolated from mice and cultured; apoptosis was induced by TNF-α and actinomycin D (Act D) for six hours. Control group was incubated with normal rat serum, while treatment group was administered with FZHY drug serum at the same time. Normal control was cultured with newborn calf serum without TNF-α and Act D. The cell apoptosis was checked with Annexin V/Propidium Iodide (PI) stain and observed under the confocal laser scanning microscopy. Early apoptotic cells were Annexin V positive (green) alone, late apoptotic and necrotic cells were both Annexin V and PI positive or PI positive (red) alone respectively. (×630).

Hepatocytic apoptosis may lead to liver fibrosis. In a recent study, we induced hepatocytic apoptosis *in vivo *through injection of Lipopolysaccharide (LPS) in mice [27]. FZHY treatment significantly attenuated hepatocytic apoptosis as indicated by terminal deoxynucleotidyl-transferase-mediated nick end-labelin (TUNEL) staining, and regulated caspase-3 activity and apoptotic factors expression in mitochondria, such as promoting the expression of anti-apoptotic Bcl-2 and counteracting the expression of pro-apoptotic Bax (Figure 8). These findings were also confirmed by *in vitro *incubation of FZHY drug serum with apoptotic hepatocytes induced by tumor necrosis factor α (TNF-α) (Figure 9).

#### Actions on sinusoidal endothelial cell and hepatic sinusoidal capillarization

Hepatic sinusoidal capillarization in endothelial space of Disse is a key event in liver fibrogenesis. Normally this space contains the components of a basement membrane that is low-dense matrix mainly consisting of type IV collagen and laminin and forms a discontinuous endothelial basement membrane. Sinusoidal endothelial cell (SEC), which lines inner sinusoid, has a lot of fenestrate. The structure of hepatic sinusoid serves as a sieve to facilitate the rapid interchange of material between the blood and hepatocytes. In early fibrogenesis, the accumulation of subendothelial matrix, in particular replacement of normally low-density matrix with high-density ones, and loss of fenestrate in SEC, would lead to transformation of continuous subendothelial basement membrane from discontinuous one and transition of closed circulation to an open one, i.e. a process known as "capillarization" [28]. Such capillarization causes hepatocytic dysfunction and high portal pressure, leading to advanced fibrosis or cirrhosis.

In our studies [29-32], the DMN-induced rats had high portal pressure and low interstitial collagenases 13 (MMP-13). The SEC damage was manifested by increased Factor VIII related antigen (vWF) expression and serum HA level. FZHY significantly improved twisting and occlusions of hepatic sinusoids, and alleviated loss of fenestrate in SEC and formation of continuous subendothelial basement membrane. FZHY decreased high portal pressure and liver fibrosis, and reduced the expression of Factor VIII related antigen and α-SMA in hepatic sinusoidal wall significantly. FZHY improved MMP-13 activity in liver tissue, through decreasing plasminogen activator inhibitor-1 (PAI-1) and TIMP-1 which are inhibitors of stromelysin and MMP-13. The results show that FZHY can inhibit and improve the reversal of hepatic sinusoidal capillarization and that FZHY's actions are associated with protection of SEC and inhibition of HSC activation.

### Coordinated effects of FZHY and its actions against liver fibrosis

The herbs in a Chinese herbal formula may have coordinated effects [33]. We used L_16 _(2^15^) orthogonal design (Table 2) and observed the coordinated effect on liver fibrosis in rats [34].

**Table 2:** Orthogonal design of FZHY pharmacological experiments L_16_(2^15^)

1	2	3	4	5	6	7	8	9	10	11	12	13	14	15
A	B	DE	C	DF			D	AD	AE	E	AF	F		CE

A: *Cordyceps*; B: *Pollen Pini*; C: *Gynostemma Pentaphyllammak*; D: *Radix Salvia Miltiorrhizae*; E: *Fructus schisandrae Chinensi*; F:*Semen Persicae*

The fibrotic models were induced by hypodermic injection of CCl_4 _plus oral administration of high fat and low protein food and DMN. The fibrotic rats were randomly divided into subgroups according to the experiment design and orally fed with different composition of herbs from start of CCl_4 _intoxication or after DMN models were established. In prophylaxis experiment with CCl_4 _model, *Semen Persicae *was a key factor to decrease hepatic hydroxyproline, *Radix Salvia Miltiorrhizae *had prominent effect on improving serum Alb and decreasing total bilirubin level, while *Cordyceps *and *Gynostemma Pentaphyllammak *had a remarkable effect on decreasing serum ALT activity. These four herbs have coordinated effects for prevention of liver fibrosis, while *Semen Persicae *and *Radix Salvia Miltiorrhizae *are the main herbs of FZHY in preventing liver fibrosis [34].

In the experiments with the DMN model of liver fibrosis, *Cordyceps *and *Gynostemma Pentaphyllammak *had strong effects on reducing hepatic hydroxyproline contents and attenuating collagen deposition, while *Radix Salvia Miltiorrhizae *and *Cordyceps Extract piece *had significant effects on improving liver function such as reducing serum ALT activity. These findings indicate that *Cordyceps *plays an important role in reversing liver fibrosis, as a main ingredient of FZHY.

While the active ingredients of FZHY have not been elucidated, we found several effective compounds from the constituent herbs of FZHY, such as amygdalin from *Semen Persicae*, salvianolic acid B (SA-B) from *Radix Salvia Miltiorrhizae*. In particular, SA-B against liver fibrosis was found effective.

### Radix Salviae Miltiorrhizae and its active ingredients

#### Action of Radix Salviae Miltiorrhizae and salvianolic acid B against liver fibrosis

In the early 1950s, there were reports on the decoctions containing *Radix Salviae Miltiorrhizae *(Sm) in treating splenomegaly due to schistosomiasis at advanced stage [35]. In recent years, this formula has been widely used to treat chronic hepatitis B and posthepatitic cirrhosis at its early stage. The extract from Sm is now used clinically as injection formulation for treating chronic hepatitis B. The liver biopsy tests before and after treatment with Sm injection revealed that liver fibrosis was improved [36]. The dynamic ultrasound Doppler examination of the patients with post-hepatitic cirrhosis revealed that Sm root injection effectively increased the portal blood flow [37].

SA-B, a major water soluble component in Sm, relieves the CCl_4_-induced fibrosis and reverses DMN-induced liver fibrosis in rats. It prevents liver cell injury, inhibits proliferation of HSCs and collagen production *in vitro *[38-42]. Therefore, SA-B is one of the active components of Sm against liver fibrosis.

**Figure 10:**
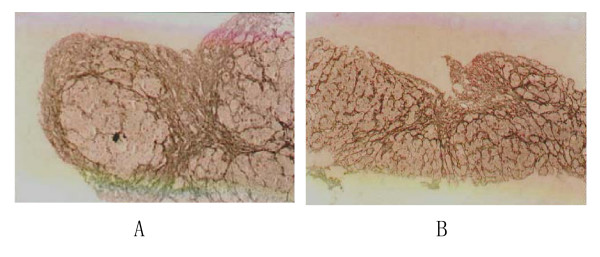
**Effect of SA-B histological changes of liver fibrosis in chronic hepatitis B**. The liver biopsy examination before treatment (A: S4) and after treatment (B: S3), stained with Gorden-Sweet and Masson trichrome method. (×100).

Between 1996 and 2001, we carried out a randomized controlled clinical trial to evaluate the clinical efficacy of SA-B in treating liver fibrosis caused by chronic hepatitis B [43]. With the randomized, double blinded and double placebo-controlled method, 60 patients with definite diagnosis of liver fibrosis caused by hepatitis B were included, and Interferon-γ (IFN-γ) was used as control drug. The patients of the treatment group orally took SA-B tablets (60 mg) or received muscular injection of IFN-γ (IMU), meanwhile the patients of the control group received placebos as injection or tablets. The complete course lasted for 6 months. The histological changes of liver biopsy specimens before and after treatment were examined together with the test results of contents of serum HA, LN, IV-C, P-III-P and liver ultrasound imaging. The results showed that both SA-B and IFN-γ treatments improve liver inflammation and fibrosis, but SA-B does better than IFN-γ. The reversal rates of fibrosis were 36.67% with SA-B and 30.0% with IFN-γ. IFN-γ treatment showed side effects such as fever, whereas SA-B treatment did not (Figure 10).

#### Actions of salvianolic acid B on TGF-β1 in hepatic stellate cell and fibrotic liver

Our *in vitro *studies [44-46] showed that 0.1 μmol/L-10 μmol/L SA-B had no toxic effect on primary cultured HSCs, but inhibited serum stimulated HSC proliferation in a dosage dependent manner as indicated by [^3^H] thymine incorporation. SA-B (1 μmol/L-10 μmol/L) had significant effects against the biological responses of TGF-β1 stimulated HSCs, including collagen gene expression, α-SMA and PAI-1 expression. Furthermore, SA-B (1 μmol/L-10 μmol/L) inhibited the plasmic and nuclear protein expression of Small Mothers against decapentaplegic deleted 2/3 (Smad2/3) and significantly inhibited intracellular phosphorylation of Smad2, decreased type I receptor expression and TβR binding. These results suggest that the main actions of SA-B against liver fibrosis are to antagonize TGF-β1-dependent activation of HSCs by inhibiting intracellular signal transduction of TGF-β1/Smads in HSCs.

## Conclusion

FZHY has been developed and tested in the past 20 years as a new Chinese medicine product to treat liver fibrosis. Although only some of the action mechanisms and active components of FZHY were discovered and much effort should be made to improve our scientific understanding, a high potential of developing new drug products such as FZHY from Chinese medicine for treating liver fibrosis has been demonstrated.

## Abbreviations

AAA: aromatic amino acid; Act D: actinomycin D; Alb: albumin; ALT: alanine; AST: aspartate aminotransferase aminotransferase; BCAA: branched chain amino acid; C3: complement 3; CCl_4_: tetrachloride carbon; KcCM: cell conditional medium; Col-I: type I collagen; D-HcCM: drug serum treated hepatic stellate cell's conditioned medium; DMN: dimethylnitrosamine; ECM: extracellular matrix; ERK: extracellular signal-regulated protein kinase; FAK: focal adhesion kinase; FN: fibronectin; FZHY: *Fuzheng Huayu*; GGT: gamma-glutamyl transferase; HA: haluronic acid; HCV: hepatitis C virus; HSC: hepatic stellate cell; Hyp: hydroxyproline; ICD-10: International Classification of Diseases, 10th edition; IFN-γ: interferon-γ; KcCM: Kuppfer cell conditional medium; LM: laminin; LPS: lipopolysaccharide; MDA: malondialdehyde; MMP-2/9: metalloproteinases-2/9; NASH: nonalcoholic steatohepatitis; PAI-I: plasminogen activator inhibitor 1; PDGF-BB: platelet-derived growth factor-BB; PI: propidium iodide; P-III-P: type III procollagen; PT: prothrombin time; SA-B: Salvianolic acid B; SEC: sinusoidal endothelial cell; Sm: *Radix Salviae Miltiorrhizae*; Smad2/3: Small Mothers against Decapentaplegic Deleted 2/3; SOD: superoxide dismutase; TIMP-1: tissue inhibitor of metalloproteinase 1; TNF-α: tumor necrosis factor α; TUNEL: terminal deoxynucleotidyl-transferase-mediated nick end-labeling; TβR-II: TGF-β type II receptor; α-SMA: α-smooth muscle actin.

## Competing interests

FZHY is a herbal product developed by the authors' institution at the Shanghai University of Traditional Chinese Medicine.

## Authors' contributions

PL and CL conceived the FZHY formula and designed the clinical trials. YYH, LMX, CHL and PL conducted the clinical trials and other experimental studies. CHL prepared the manuscript. All authors read and approved the final version of the manuscript.
